# Nutrition Knowledge, Attitude and Practice of College Sportsmen

**DOI:** 10.5812/asjsm.34866

**Published:** 2010-06

**Authors:** Peerkhan Nazni, Srinivasan Vimala

**Affiliations:** 1 Department of Food Science, Periyar University, Salem, Tamilnadu, India; 2 Department on Nutrition, Shanmuga college of Nursing, Salem, Tamilnadu, India

**Keywords:** Athletes, Practice, Supplements, Knowledge, Attitude

## Abstract

**Purpose:**

Nutrition is an important component of any physical fitness program. The main dietary goal for active individuals is to obtain adequate nutrition to optimize health fitness and to increase sports performance. The present study aims to assess the nutrition knowledge, attitude and practice among the selected athletes.

**Methods:**

Athletes from five different private colleges situated in Salem District, Tamilnadu, India were selected. A total number of 102 athletes, 32 sportsmen belong to Volleyball discipline, 25 belongs to weightlifter discipline and 45 belong to runners discipline in sports. All the selected athletes were including in the study. The Knowledge, Attitude and Practice (KAP) questionnaire contained ten questions about nutrition knowledge, nine questions about attitudes, and ten questions about dietary practice were collected from the selected athletes. Dietary composition of the sportsmen is also assessed. The collected data was coded and used for evaluation.

**Results:**

Results about KAP revealed that 42 per cent of the volleyball players had good nutritional knowledge (60–69per cent) compared to weight lifters (43per cent) who had satisfactory (50–59per cent) knowledge about nutrition. Twenty nine per cent of the runners had very good (70–79per cent) knowledge about nutrition. Regarding food consumption pattern intake of cereals, other vegetables and milk was found to be less compared to the RDA for the athletes. Among the three disciplines sports persons, the mean nutrient intake of the runners is high compared to volleyball and weight lifters.

**Conclusion:**

The sports disciplines strongly affected the nutrition knowledge, attitudes and practices of sportsmen. The overall scores indicate that most sportsmen had good knowledge of nutrition and supplements.

## INTRODUCTION

Nutrition plays a very important role in attaining high level of achievements in sports. Nutritional status has a direct bearing on the level of physical performance. Hence, physical fitness and training are very much dependent on nutritional status of sports personnel^[1]^. Nutrition is an important complement of any physical fitness program. The main dietary goal for active supplement information from nutritionists/dietitians and individuals is to obtain adequate nutrition to optimize health and fitness or sports performance^[2]^.

Nutrition is an important component of any physical fitness program. The main dietary goal for active individuals is to obtain adequate nutrition to optimize health and fitness or sports performance^[3]^. This is not only important to help to improve performance but also to promote healthy dietary practices in the long term^[4]^. So, a reasonable strength and condition program and a well balance diet must be presented as a sensible alternative to a riskier, shortcut mindset^[5]^.

Compare with parents, it has been found that trainers had more influence on the attitude, subjective norms, and intention of adolescents regarding supplement use^[6]^. Adolescents from low income communities receive less educational resources and may possess insufficient knowledge of nutrition and sports supplements to make health conscious decisions. Their study also indicated that a short – term nutritional education program can significantly improve supplementations knowledge^[7]^.

However, there have been few studies assessing nutritional knowledge, attitudes and practices (KAP) of male athletes. The purpose of this study was to assess the dietary composition and nutrient intake and compare with RDA and also to evaluate the nutritional knowledge of the selected sportsmen.

## METHODS AND SUBJECTS

**Subjects:** The study cohort, 102 sportsmen belongs to five private colleges affiliated to Periyar University, Salem, Tamilnadu, India with the age group of 19–24 years was selected by purposive sampling techniques. The subjects in the present study were state and national sportsmen. The sportsmen belonged to varied sports disciplines viz. volleyball (n=32) weight lifters (n=25) and runners (n=45). Letters requesting trainers consent were sent to their homes or clubs of the respective sportsmen. The study had been approved by the Ethical Committee Members of the institution.

**Questionnaire development:** A well framed and standardized questionnaire cum interview schedule was used to elicit information on the following aspects. It consists of questions under various concepts such as demographic, nutritional knowledge, attitudes, practices and sportsmen supplements questions. This question was developed from a similar study evaluating the nutritional knowledge of athletes by Mitchell^[8].^ Face and content validity of the questionnaire was established in the study. The content of this questionnaire was based on the common practices followed by south Indian athletes.

The diet recall was a documentation of the actual application of the selected player's nutritional knowledge. It consisted of 15 multiple choice questions regarding particular foods that were consumed with the last seven days. The diet recall contains questions that were a modification of the nutritional section of the 2005 Youth Risk Behavior Survey that is completed each year by the Center for Disease Control Prevention^[9]^. The diet recall answers were based on the 1996 food guide pyramid, since the participants obtained information from health class using the previous guidelines. One registered and licensed dietitian examined the questionnaire and diet recall for clarity, order and selections as well as for content and face validity. The diet recall was also viewed by five certified athletic trainers who have nutrition knowledge and experience working with athletes from various disciplines.

The developed interview scheduled was coded and used for evaluation. The dietary composition and nutrient intake such as energy, protein, carbohydrate, fat, iron and calcium was calculated using the nutrient value of Indian Food ^[10]^ and compared with RDA. The entire data was collected over a period of six months from January 2007 to June 2007.

**Procedures:** Questionnaires were distributed to the selected athletes. A cover letter was included to explain the purposes of the study to the participants and to indicate their rights as participants. The demographic questions, nutritional knowledge questionnaire and diet recall were distributed to the athletes on an assigned day. The cover letter and directions were read aloud while the participants read along. After the direction was read, time was allotted for the participants to complete the study. After completing both questionnaires and the diet recall, the participants were asked to place them in a labeled envelope in the front of the room. The primary investigator collected the envelope after the participants left the room.

**Statistical analysis:** Pearson correlation and Z scores were used to analyze the KAP. Z scores are used to establish how many standard deviations above or below the mean test score value lies. All statistical analysis was performed with the use of SPSS for WINDOWS (version 15; SPSS Inc, Chicago).

## RESULTS

**Demographic information:** The results showed that 55% of the selected athletes were in the age group of 20±0.2 years and 45% of them belongs to the age group of 23±0.6 years. All the participants were males. About 60.7% of the participants were in Undergraduate degree course and 39.3% of the participants were doing their Post graduate degree courses. Regarding the average number of years the participants played their respective disciplined sports revealed that 66.3% of the participants were involved for 20.7±0.6 years and 33.7% of them were involved for 5.07±1.57 years. Seventy eight percent of the participants said they learned about nutrition in health class. About 80% of the participants ate breakfast on most days. There were 95% of the participants who ate their lunches every day. Result about frequency of meals and snacks consumed by the participants indicated the average meals were 3 meals and the average numbers of snacks were 4 snacks per day.

**Food Intake:** Table 1 presents the composition of the diet consumed by the sportsmen studied. The comparison of the food ingredients recommended in diet A 1993 indicate that there was a vast difference in the actual consumption. The intake of cereals and other vegetables was less among all the participants. The milk and fat consumption of Runners was high. The consumption of fruits and vegetables was always stressed for their potential health benefits however the recommended scale of ration is low.

**Table 1:** Dietary Composition of sportsmen

Nutrients	Recommended dietary allowances CES 1993 g/day/person	Volley ball players	Weight lifters	Runners
**Cereals**	550	340	340	348
**Pulses**	40	78	55	55
**Protective vegetables**	150	260	200	200
**Other vegetables**	200	130	90	110
**Roots and tubers**	150	200	180	180
**Fruits**	150	410	300	300
**Milk**	1000	760	800	1400
**Fat**	50	60	65	67
**Sugar**	75	80	55	55
**Meat**	400	425	460	510
**Eggs**	100	125	100	100

Regarding diet recall response (Table 2) 4.58% of the participants drank 100% fruit juice 4– 6 times in the last seven days. During the past seven days 26.79% of the participants ate fruit 4–6 times. About 36.59% of the participants ate carrot 4– 6 times in the past seven days. About 43.79% of participant's drank milk 3 times per day. Fast food was preferred by 25.49% of the participants one time per day. About 35.95% of the participants drank only 4– 6 times of soda during the past seven days. Regarding junk food about 24.83% of the participants consumed 2 times per day. Results on water consumption revealed that 37.25% of the participants either drank water 4– 6 times in the past seven days, 2 times (17.65%), 3 times (5.88%) or 4 times (19.61%) per day.

**Table 2:** Diet recall response of the sportsmen

Frequency consumption of foods for past 7 days	Did not	1–3 times during the past 7 days	4–6 times during the past 7 days	1 time per day	2 times per day	3 times per day	4 times per day
**100per cent fruit juice (200ml)**	20.1	3.26	4.58	11.76	9.80	17.64	11.10
**Fruit (1 medium size)**	–	12.42	26.79	13.06	9.15	12.42	24.18
**Salad(50g)**	28.43	10.46	–	3.92	24.19	29.41	11.11
**Potatoes(50g)**	11.76	17.65	20.26	10.46	16.99	16.99	7.84
**Carrots(50g)**	11.11	15.03	36.59	6.54	18.30	–	10.46
**Other vegetables (50g)**	4.08	9.15	23.53	30.71	10.46	15.69	3.92
**Glass of milk (150ml)**	–	7.19	9.80	19.60	11.76	43.79	5.88
**Chicken, fish or red meat (200g)**	5.23	35.29	20.92	26.15	10.46	5.23	–
**Beans, peanut, butter, peas and nuts (each 100g)**	–	24.19	5.23	26.15	17.64	15.02	11.76
**Cereals or bread (500g)**	9.15	24.19	7.19	28.11	17.65	13.73	11.76
**Rice, pasta and crackers (500g)**	18.95	9.80	3.26	39.22	4.57	13.73	8.5
**Fast foods (200g)**	4.58	12.42	3.26	25.49	17.64	2.77	7.19
**Glass of soda(cont one 12oz can of soda or a 16oz bottle as one glass)**	–	11.11	35.95	14.38	8.5	15.03	13.07
**Junk foods (150g)**	14.05	9.80	11.11	24.19	24.83	6.54	6.54
**Glasses of water (150ml)**	–	11.11	37.25	6.54	17.65	5.88	19.61

**Nutrient Intake:** Nutrient intake of elite athletes is a critical determinant of their performance and ability to compete. The nutrient intake (Table 3) shows significant variation with respect to sports discipline and body weight. The nutrient intake of the sportsmen was well comparable with RDA expect for protein and iron.

**Table 3:** Nutrient intake of south Indian sportsmen

Category	Body wt (kg)	Energy (Kcals)	Protein (g)	Carbohydrate (g)	Fat (g)	Iron (g)	Calcium (g)
**Volley ball players**	55±6.7	3972.6±575.6	122.2±11.5	485.5±50.9	109.4±17.6	25±4.21	1650±105.3
**Weight lifters**	69.1±8.21	3563.7±350.7	155.3±30.2	545.1±82.4	113.5 ±13	35.6±4.03	1810±238.2
**Runners**	60.2±5.4	4330.6±675.6	114.3±21.5	480.5±120.0	107.8±17.6	28.6±3.2	1440±151.2
**RDA**		3000–6000	135–225	400–600	120–200	50	1500–2000

Ref: Sathyanarayana et.al (1985) ^[11]^

The calorie intake was positively correlated with the body weight (r = 0.5). The protein intake was low in comparison to RDA for Volleyball players and Runners. Both fat and iron intake was also low in comparison to RDA for volleyball players, weight lifters and runners.

**Nutrition Knowledge:** Elite athletes are generally knowledgeable and sophisticated with regard to nutrition and effect on performance. Runners were best informed and aware about the role of nutrients in athletic performance followed by volleyball players and in that order with weight lifters faring least score. Carbohydrate was reported to be the concentrated source of energy followed by protein and fat. Sportsmen were aware of the role of protein in muscle building. The runners had a better knowledge about the role of vitamins and minerals and were able to list the sources of iron, calcium, vitamin A and vitamin C and the protective effects of fruits and vegetables. The sportsmen were aware of the importance of hydration during the athletic period (Table 4).

**Table 4:** Nutrition knowledge of sportsmen

S.No	Nutrition Aspects	Volley Ball players (positive answers%)	Weight lifters (positive answers%)	Runners (positive answers%)
**1**	**Carbohydrate**	56	68	79
**2**	**Protein**	42	43	75
**3**	**Fat**	35	18	39
**4**	**Vitamins and minerals**	45	23	48
**5**	**Hydration**	51	17	43

**Nutritional Attitude and Practice:** The statements designed for the attitude test were based on certain common beliefs, tradition, food fads and attitudes of Indian athletes (Table 5).

**Table 5:** Attitude of sportsmen regarding nutritional practices

S. No	Statements	Correct Response	Percentage correct
1	A lack of Iron in the diet can result in fatigue, injury and illness	SA	53.3
2	Dehydration can impair physical performance	SA	72
3	The Nutritional needs of athletes differ from normal population	SA	60
4	Vitamin supplementation is recommended for physically active people	SA	80
5	Excess vitamin supplementation may harm the physically active person	SA	76.66
6	High consumption of ghee almonds and milk enhance performance	SA	46.66
7	High amount of water consumption will increase the body weight	SD	86.66
8	Dietary pattern should change with season and climatic conditions	A	65.33
9	How does exercise diet protein requirement	A	90

SA: Strongly agree/A: Agree/U: Undecided/D: Disagree/SD: Strongly Disagree

About 90% of sportsmen agreed the attitude about exercise affect protein requirement, 87% of them strongly disagree the statement of high water consumption will increase the body weight, 80 percent of them strongly agree that vitamin supplementation is recommended for physically active people and 72% of the athletes strongly agree that dehydration will impair physical performance. Only 46.66% of them had a correct attitude that high consumption of ghee, almonds and milk enhance the performance of the athletes.

The dietary practices (Table 6) of the sportsmen were variable. Among the selected athletes 63% of them adopted a change in the dietary pattern at the time of competition. Skipping of meals prior to competition was participated by 69 percent of the selected athletes. Awareness about carbohydrate loading was seen in 38 percent of the selected athletes and consumption of glucose polymer drink during exercise was followed by 58 percent of the athletes. Habit of taking energy bars during exercise was practiced by 34 percent of the athletes, consumption of energy gel during exercise was not observed in 64 percent of the athletes, consumption of rising was practiced by 66 percent of the athletes at the time exercise. Habit of consuming isotonic sports drinks was observed in 44 percent of the selected athletes.

**Table 6:** Dietary practices of sportsmen

S. No	Questionnaires	Yes	No
		No (%)	No (%)
**1**	Is you dietary pattern change at the time of competition	95 (63.3)	55 (36.67)
**2**	Do you skipping meals prior to competition	47 (31.33)	103 (68.67)
**3**	Do you consuming sports drinks every day before practicing	39 (26)	111 (74)
**4**	Do you practice carbohydrate loading prier to competition	58 (38.67)	92 (61.33)
	Do you consume glucose polymer drink (12g of glucose/100ml) during exercise?	87 (68)	63 (42)
**5**	(A) 250 ml (30g CHO)	A=44	
	(B) 500 ml (60g CHO)	B=43	
	Are you having the habit of taking energy base during exercise?	51 (34)	99 (66)
**6**	(A) ½–1 bar (30g CHO)	A=24	A=38
	(B) ½–1 bar (60g CHO)	B=27	B=61
	Do you consume energy gel during exercise	54 (36)	96 (64)
**7**	(A) 1 sachet (30g CHO)	A=20	
	(B) 2 Sachet (60g CHO)	B=34	
	Will you consume rising (or) sultanas at the time exercise?	99 (66)	51 (34)
**8**	A) 40g (30g CHO)	A=54	
	B) 80g (60g CHO)	B=45	
	Will you practice of eating bananas during exercise?	87 (68)	63 (42)
**9**	a) 1–2 banana (30g CHO)	A=44	
	b) 2–3 bananas (60 g CHO)	B=43	
	Do you consume isotonic sports drink (6g/100ml) during exercise	66 (44)	94 (36)
**10**	A) 500 ml (30g CHO)	A=23	
	B) 1000 ml (6g CHO)	B=43	

Sports discipline had strong influence on the nutrition knowledge, attitudes and practice of selected sportsmen. The interview schedule used to gather information about nutrition Knowledge, Attitude and Practice (KAP) was coded and analyzed using ‘Z’ score (Table 7). Z scores help to establish how many Standard Deviation (SD) lies above or below the mean test score values. The overall scores indicate that most sportsmen had satisfactory knowledge of nutrition and supplements.

**Table 7:** Nutrition knowledge, Attitudes and practice of sportsmen based on Z Scores

Standard scores	Volley ball players (%)	Weight lifters (%)	Runners (%)
**Excellent > 80**	Nil	14	3
**Very good 70–79**	18	7	29
**Good 60–69**	42	21	32
**Satisfactory 50–59**	25	43	25
**Regular 40–49**	7	11	7
**Poor 30–39**	8	4	4
**Very poor 20–29**	Nil	Nil	Nil
**Bad < 20**	Nil	Nil	Nil

## DISCUSSION

Breakfast as a part of a healthy diet and life style can positively impact student's health and well being. Compared to 80% of the participants in this study who ate breakfast, Buergel's^[12]^ study indicated that only 41 per cent eat breakfast, while the remaining 59% skip breakfast more than three times a week. Hickson^[13]^ study indicated that only 19 per cent of his sportsmen ate breakfast and 81 per cent skipped breakfast almost daily. Although in one study 87% of participants said they ate breakfast on most days, 54% of those breakfast were high in fat^[14]^. By skipping breakfast, adolescents have already started off poorly and are mission out on the essential nutrients^[15]^. Regarding diet recall responses of the athletes about 43.79% of the athletes drank 3 cups of milk 3 times every day. This is very similar when compared to the new My Pyramid guidelines in which 3 cups are recommended every day.

Calcium intake is an important source of growing strong healthy bones. Not getting proper amounts of calcium in the diet can pose a big health risk for adolescent's athletes now and in the future.

Athletes are not getting the recommended serving of fruit and bread cereals group. Only 9.15% of participants eat My pyramid recommended 2 cups of fruits per day^[16]^. The serving size is similar to the 1996 Food Guide Pyramid of 2–4 serving per day consisting of 1 medium piece of fruit or ½ cup of chopped fruit^[17]^. Around 10.46% of participants are consuming the recommended serving of meat or protein. My pyramid recommends that adolescents consume 5.5 oz meat daily^[16]^. This is similar to the 1996 Food Guide Pyramid's recommendations of 2–3 serving (App. 200g) of chicken or beef per day^[17]^.

There were encouraging results from this study. Results about fast food consumption revealed that 25.49% of the participants ate fast food only once a day and 4.58% of the participants did not eat in the past seven days. These numbers are surprising considering the prevalence of fast food in today's world. Eating fast food only one time in a week is not a poor choice when considering that some fast food choices may actually be healthy. One study indicated that 77.5% of participants ate junk food daily and the majority consumed junk food several times a day^[18]^.

Results about soda consumption indicated that the participants are not drinking soda in excess. About 14.38% of the participants drank soda only one time in the last week. These numbers seem low to one study that claimed that 79.4% of adolescents consume soft-drinks daily^[19]^.

## CONCLUSION

The present study reveals that there is a paucity of nutrition education intervention among selected sportsmen. There is sports specific variation in the food fads and practices indicating the strong influence of coaches and peers and tradition. It is vital to educate the sportsmen and accustom them to dietary pattern in different regions of India and abroad. Failure to consume right diet during competition due to false belief and constant fear of eating prohibited foods may hamper performance. Hence, delivering continuous education through workshops and courses helps to improve trainers’ nutritional knowledge, attitudes and practices.

## References

[1] Beals KA, Manore MM (1998). Nutritional status of female athletes with subclinical eating disorders. J Am Diet Assoc.

[2] Congeni J, Miller S (2002). Supplements and drugs used to enhance athletic performance. Pediat Clin North Am.

[3] Berning JR, Mahan KL, Escott-Stump S (2000). Nutrition for Exercise and Sports Performance. Krause's Food. Nutrition and Diet Therapy.

[4] Jonnalagadda SS, Rosenbloom CA, Skinner R (2001). Dietary practices, attitudes, and physiological status of collegiate freshman football players. J Strength Cond Res.

[5] Weber (2004). Validity of self-reported dietary intake at school meals by American Indian children; the pathways study. J Am Diet Assoc.

[6] Dunn MS, Eddy JM, Wang MQ (2001). The influence of significant others on attitudes, subjective norms and intentions regarding dietary supplement use among adolescent athletes. Adolescence.

[7] Little JC, Perry DR, Volpe SL (2002). Effect of nutrition supplement knowledge among high school students from a low-income community. J Comm Health.

[8] Mitchell C (2004). Nutritional knowledge of high school athletes. West Virginia University school of Physical Education Thesis.

[9] Shier F (1995). High school athletes and nutritional supplements; A study of knowledge and use. Int J Sport Nut.

[10] Gopalan C, Sastri R, Balasubramanian SC (2000). Nutritive Value of Indian Foods.

[11] Stang M (2002). Relationships between vitamin and mineral supplement use, dietary intake and dietary adequacy among adolescents. J Am Diet Assoc.

[12] Buergel NS, Bergman EA Students consuming snack, lunches devote more time to eating than those consuming school lunches. J Am Diet Assoc.

[13] Hickson JF, Duke MA, Risser WL (1987). Nutritional intake from food sources of high school foot ball athletes. J Am Diet Assoc.

[14] Schmal Z (1993). Nutritional beliefs and practices of adolescents athletes. J Sch Nurs.

[15] Voget M, Puntschart A, Howard H (2003). Effect of dietary fat on muscle substrates, Metabolism and Performance in Athletes. Med Sci Sports Exerc.

[16] Loud K (2003). Primary care of the elite-emulating adolescent male athlete. Adolesc Med.

[17] Macdermid PW, Stannard P (2006). Whey supplemented high-protein diet versus a high-carbohydrate diet. Int J Sports Nut Exercise Metab.

[18] Frary CD, John Son RK, Wang MQ (2001). Children and adolescents choices of food and beverages high in added sugars are associated with intakes of key nutrients and food groups. J Adolesc Health.

[19] Werblow JA, Fox HM, Henneman A (1978). Nutritional knowledge attitudes and food patterns of women athletes. J Am Diet Assoc.

